# Establishment and maintenance of sexual preferences that cause a reproductive isolation between medaka strains in close association

**DOI:** 10.1242/bio.022285

**Published:** 2017-02-08

**Authors:** Mayuka Ikawa, Emi Ohya, Hiroka Shimada, Makiko Kamijo, Shoji Fukamachi

**Affiliations:** Department of Chemical and Biological Sciences, Japan Women's University, Mejirodai 2-8-1, Bunkyo-ku, Tokyo 112-8681, Japan

**Keywords:** Mate choice, Medaka, Somatolactin alpha, Skin color, Sympatric speciation

## Abstract

Animals choose reproductive partners based on their sexual preferences which are established at a certain time point before, during, or after sexual maturation. The preferences are often divergent within a species, which suppresses gene flow between populations and may promote speciation. There are two strains of medaka (*Oryzias latipes*) that differ by a single transgene and mate assortatively depending on skin color. Here, we demonstrate that symmetrically biased (mutually exclusive) sexual preferences are (1) gradually established during growth depending on skin color and the color of surrounding fish, (2) strong enough to minimize gene flow between the strains at a population level, and (3) inflexibly retained after sexual maturation, even after weeks of daily mating with partners of the other strain. Thus, these laboratory strains of medaka are under premating isolation with the simplest genomic structure. They provide an empirical platform for assessing the complex and hypothetical mechanisms of speciation by mate choice.

## Abstract (precis)

**Summary:** Two laboratory strains of medaka assortatively mate in complete sympatry, and their sexual preferences are gradually developed during growth and firmly maintained after sexual maturation.

## INTRODUCTION

Mate choice or selecting reproductive partners is one of the most active fields of research in animal biology, and many studies have been conducted using various species for decades (Jones and Ratterman, 2009; Scordato et al., 2014). Secondary sexual characteristics, such as color, ornaments, dances, songs, and pheromones, often appear in either sex to attract the other and are divergent among species, demonstrating that different animals choose mates based on different criteria. Generally, males are more exaggerated and less choosy than females, and research tends to focus on mate choice by females (female choice); however, there is increasing evidence for male choice (Avigliano et al., 2016; Bloch et al., 2016; Utagawa et al., 2016) showing the importance of sexual traits in females to trigger the courtship behavior of males.

The diversity of sexual traits, and therefore that of sexual preferences, often exists among closely related species, or even within a species, and is hypothesized to suppress gene flow between polymorphic populations and promote speciation by the Fisherian runaway process (Higashi et al., 1999; Ortiz-Barrientos and Noor, 2005). Importantly, such hypotheses all assume that sexual preferences are genetic traits that are relatively unaffected by postnatal experiences, otherwise sympatric animals experiencing identical environments would establish the same preferences. However, the contribution of heritable factors to sexual preferences was shown to be low in at least some species (Schielzeth et al., 2010).

In contrast, many studies have demonstrated that social or sexual experiences, such as fostering, cohabitation, mating, etc., have significant effects on sexual preferences (Iyengar et al., 2002; Williams et al., 1992; Yamazaki et al., 1988); i.e. sexual preferences are not genetic but acquired traits. However, the preferences observed in such studies were often weak, leaving serious doubts that biased sexual preferences alone may not be sufficient to suppress gene flow (Arnegard and Kondrashov, 2004; Kirkpatrick and Nuismer, 2004; van Doorn et al., 2004). It is also a problem that the weakness of preferences allows only descriptive or theoretical studies and will not provide an empirical platform to delve into genetic or physiological mechanisms for mate choice.

From these standpoints, medaka (*Oryzias latipes*) could be an ideal model. This fish has a long history of classical genetics (Aida, 1921), inbred strains are available (Hyodo-Taguchi and Matsudaira, 1984), its genome sequence has been read (Kasahara et al., 2007), genome editing techniques are applicable (Ansai and Kinoshita, 2014), and the edited genomes can be stored as frozen sperm (Yang et al., 2010). Its sexual cycle is a single day (i.e. females spawn every day), mating behaviors have been well documented (Kinoshita et al., 2009) even in the state of zero-gravity (Ijiri, 1995), neurons responsible for female choice of familiar males have been identified (Okuyama et al., 2014), and there are laboratory strains that mate assortatively (Fukamachi et al., 2009a).

The laboratory strains that mate assortatively are a mutant of the *somatolactin alpha* (*SLα*) gene, called *color interfere* (*ci*) (Fukamachi et al., 2004), and a transgenic *ci* that overexpress *SLα* under the regulation of the *beta-actin* promoter, called Actb-SLα:GFP (Fukamachi et al., 2009b). Of the four types of pigment cells in medaka skin, light-reflecting ones (white leucophores and silvery iridophores) are increased in *ci* causing a pale skin, whereas light-absorbing cells (orange xanthophores and black melanophores) are increased in Actb-SLα:GFP causing a dark skin. Males of both strains explicitly prefer to mate with females of the same strain. For example, a male that was given a choice between two females of the same and different strains in a 1.3-liter tank completely ignored the latter, while courting the former over 80 times in an hour (Fukamachi et al., 2009a).

These strains could be a tool for investigating mechanistic structures for mate choice (e.g. the establishment of preferences, perception of traits, and decision of mating), which may provide a hint in understanding the complex sexual selection that occurs in the field. Based on our previous study (Utagawa et al., 2016), we further characterized the symmetrically biased sexual preferences of *ci* and Actb-SLα:GFP males, focusing on their stability (Experiment I), strength (Experiment II), and establishment (Experiment III).

## RESULTS

### Experiment I: the stability test

**Fig. 1.**
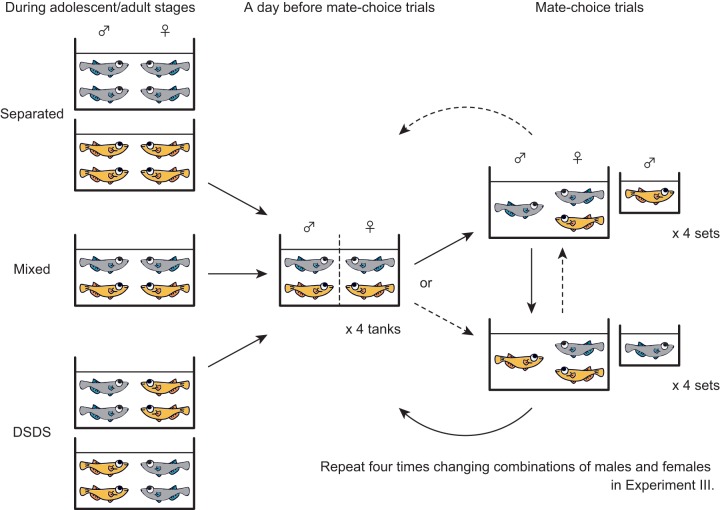
**Brief overview of the fish breeding and mate-choice trials.** During larval/juvenile stages, we kept fish under separated or mixed conditions (not shown in this figure). During adolescent/adult stages (left), we put four or more fish into a tank and kept them under either separated, mixed, or different-sex-different-strain (DSDS) conditions. On the day before mate-choice trials (middle), we prepared four tanks using 16 fish (eight males and eight females) by putting one *ci* male, one Actb-SLα:GFP male, one *ci* female, and one Actb-SLα:GFP female into each tank, and kept different sexes in different compartments using a separator (dotted line). On the day of the mate-choice trials (right), we took one male out from each tank and performed the first trials. Then, we exchanged the males and performed the second trials. In Experiment III, we repeated the trials four times on four consecutive days by rotationally putting the males into different tanks.

**Fig. 2.**
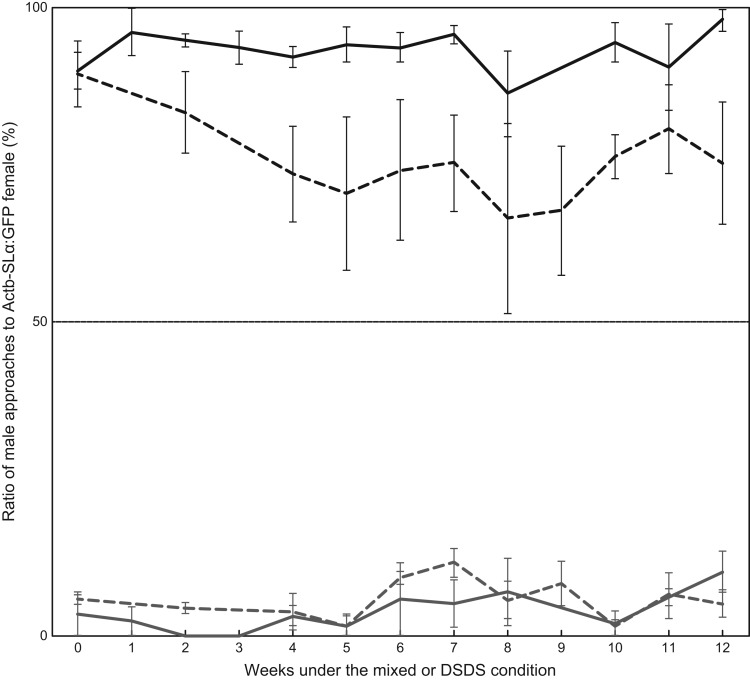
**Results of Experiment I, the stability test.** Mature males of *ci* (gray lines) and Actb-SLα:GFP (black lines) were kept in the mixed (solid lines) or different-sex-different-strain (DSDS, dotted lines) conditions for 12 weeks (*n*=4 per strain per condition), and given a choice once a week between *ci* and Actb-SLα:GFP females. Preferences of the males (mean and s.e.m.) are shown as the ratio of approaches to Actb-SLα:GFP females. Data for the ninth week in the mixed condition and the first and third weeks in the DSDS condition are missing. Note that the males never preferred females of the different strain. Seventeen out of 184 trials (2 strains×2 conditions×4 trials×13 weeks – 24 missing trials) were invalid (i.e. less than 10 approaches per trial) and we excluded the data from the analyses.

We placed matured males of *ci* and Actb-SLα:GFP (*n*=8 for each strain), which had preferred females of the same strain (‘Week 0’ in Fig. 2), in either of the following conditions for 12 weeks: (1) the mixed condition where one *ci* male, one Actb-SLα:GFP male, one *ci* female, and one Actb-SLα:GFP female were kept in a tank (four sets), or (2) the different-sex-different-strain (DSDS) condition in which two *ci* males and two Actb-SLα:GFP females, or two Actb-SLα:GFP males and two *ci* females, were kept in a tank (two sets each; see Fig. 1). During breeding, we tested sexual preferences of each male once a week. This means that we measured sexual preferences of four males from each strain and condition every week.

Medaka spawn every morning, and mating between *ci* and Actb-SLα:GFP did occur under the DSDS conditions (see Experiment IIb) although they are mutually unattractive (Fukamachi et al., 2009a; Utagawa et al., 2016). That is, males in the DSDS condition mated daily with females of the different strain for 12 weeks. However, the males in either of the mixed or DSDS conditions never preferred females of the different strain (Fig. 2). Even alleviation of the biased preferences seemed not to occur.

### Experiment IIa: the strength test (two-male-one-female trials)

We quantified the degree of sexual isolation between *ci* and Actb-SLα:GFP in two ways (Experiments IIa and IIb). First, we questioned why *ci* and Actb-SLα:GFP males seldom court females of different strains (Fig. 2) (Fukamachi et al., 2009a; Utagawa et al., 2016). To address this question, we observed mating behaviors under the condition in which a pair of *ci* and Actb-SLα:GFP males was presented with one *ci* or one Actb-SLα:GFP female. Body lengths of the competing males were not identical; among the male pairs tested (*n*=4), *ci* was larger than Actb-SLα:GFP in three of the four pairs.

**Fig. 3.**
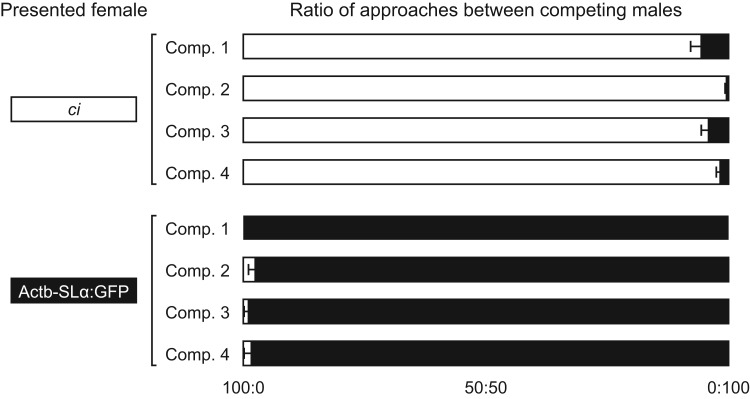
**Results of Experiment IIa, two-male-one-female trials.** Either a *ci* or Actb-SLα:GFP female (left) was presented to a competing pair of *ci* and Actb-SLα:GFP males (Comp. 1∼4). Each horizontal bar shows an average ratio of male approaches in each competition (white, approaches from *ci*; black, approaches from Actb-SLα:GFP). Error bars are s.e.m. There was no invalid data in a total of 32 trials (4 competing male pairs×2 presented females×4 trials); i.e. 10 or more approaches could be counted in all the trials.

We presented to each male pair a total of eight females (four *ci* and four Actb-SLα:GFP) on four consecutive days (one *ci* and one Actb-SLα:GFP female per day). In all the 32 trials (four male pairs×eight females), one of the males extensively courted a female, whereas the other male seldom or never did. It was apparent that this was not because of a hierarchical relationship between the males because courting males changed when the females presented were changed. All males extensively courted only when a female of the same strain was presented (Fig. 3). Thus, the males seemed to have little interest in females of the different strain.

### Experiment IIb: the strength test (paternity identification after mass breeding)

Next, we hypothesized that the symmetrical, strong, and long-lasting sexual preferences (or sexual indifferences) of *ci* and Actb-SLα:GFP (Figs 2 and 3) may suppress gene flow between these conspecific and post-zygotically compatible strains. To test this, we put six males and six females in a tank, collected eggs every morning, and identified the paternity of each embryo by GFP fluorescence.

We first put six *ci* females and six Actb-SLα:GFP males in a tank for five days. Four to six females spawned daily, but the spawning often started late in morning (about 10:00 h); for instance, one female spawned in the afternoon on the first day. We obtained a total of 101 fertilized eggs and all the embryos were, needless to say, GFP-positive (Trial I in Table 1).

**Table 1.**
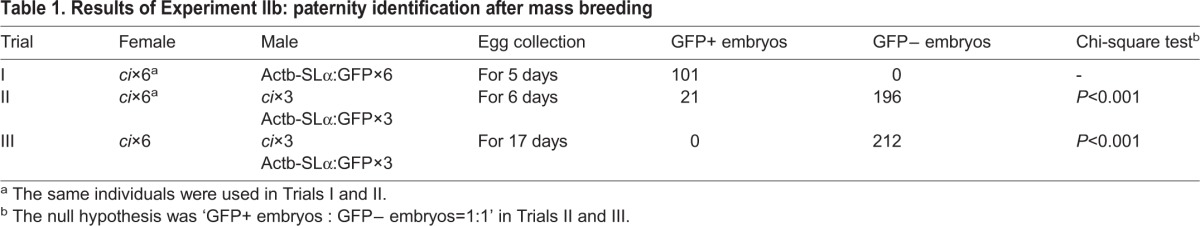
**Results of Experiment IIb: paternity identification after mass breeding**

We then replaced the six Actb-SLα:GFP males with three *ci* males and three Actb-SLα:GFP males and continued collecting eggs for another six days. Similar to Trial I, four to six females spawned daily, but all spawning was usually completed by 09:00 h. We obtained a total of 217 fertilized eggs, 21 of which were GFP-positive, demonstrating that the *ci* females produced 9.3-fold more offspring with the *ci* males than they did with the Actb-SLα:GFP males (Trial II in Table 1).

We repeated Trial II using different individuals of males and females. Because the new *ci* females spawned a fewer number of eggs, it took 17 days to collect 212 fertilized eggs. Nonetheless, none were GFP-positive, demonstrating that the females never reproduced offspring with the Actb-SLα:GFP males (Trial III in Table 1). Thus, a gene flow from Actb-SLα:GFP males to *ci* females is strongly (Trial II) or completely (Trial III) suppressed, even when the strains were in close contact in a small tank. We assume that this is largely because of the sexual indifference of Actb-SLα:GFP males to *ci* females (Fig. 3), but do not exclude a significant contribution from female choice, i.e., rejection of spawning by the females.

A gene flow in the other direction, from *ci* males to Actb-SLα:GFP females, cannot be assessed by this method because all eggs that Actb-SLα:GFP females spawn will become GFP-positive, irrespective of paternity; however, we suspect that it would also be suppressed considering the symmetrical characteristics of sexual preferences between *ci* and Actb-SLα:GFP medaka (Figs 2 and 3).

### Experiment III: the establishment test

The only difference between genomes of *ci* and Actb-SLα:GFP medaka is the *SLα* transgene (Fukamachi et al., 2009b). Hence, this transgene must be the cause of their symmetrically biased sexual preferences. However, this does not necessarily mean that the preferences are genetic traits because the fish used in the previous and present studies had been reared under separated conditions; different strains had experienced different breeding conditions prior to the mate-choice trials.

Thus, there seemed to be three possible, not necessarily alternative, mechanisms in which the *SLα* transgene biases sexual preference: (1) SLα acts directly on the brain and its low or high expression makes medaka prefer *ci* or Actb-SLα:GFP, respectively (i.e. preference is a genetic trait); (2) SLα controls only the skin color and medaka prefer fish with the same color (i.e. preference is a genetic but acquired trait); and (3) SLα controls only the skin color and medaka prefer the color of fish that they were reared with (i.e. preference is an acquired trait).

**Fig. 4.**
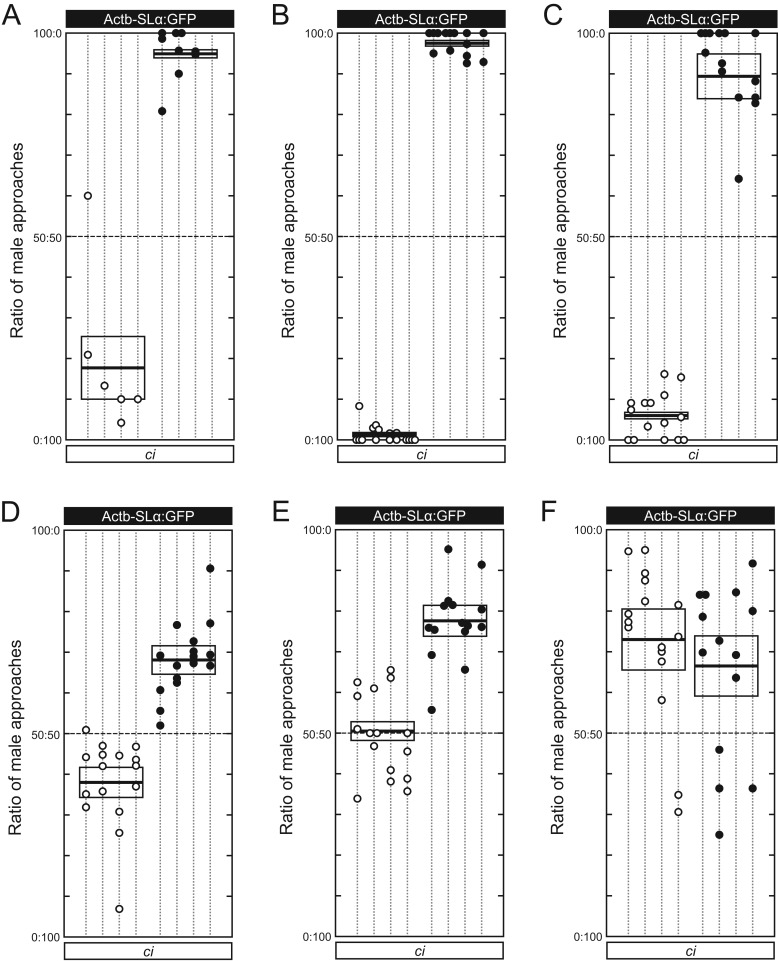
**Results of Experiment III, the establishment test.** In each figure, presented females are shown on top (Actb-SLα:GFP) and bottom (*ci*), and each male is represented by a dotted vertical line (*n*=4 per strain per condition). Four mate-choice trials were performed using each male, and the ratios of approaches between the presented females are shown as four circles on each line (white, *ci*; black, Actb-SLα:GFP). Missing circles indicate invalid data (e.g. the right-most Actb-SLα:GFP male in A approached females less than 10 times in all the four trials, and therefore there is no circle on the dotted line). A box with a horizontal line in the middle indicates a mean and s.e.m. (A) Separated conditions for the first two months and then mixed conditions afterwards. (B) Separated conditions for the first two months and then different-sex-different-strain (DSDS) condition afterwards. (C) Mixed conditions for the first two months and then separated condition afterwards. (D) Mixed conditions. (E) Mixed conditions for the first three months (high fish densities suppressed the speed of growth and maturation), and then DSDS condition afterwards (until five months old). (F) Mixed condition for the first two months, and then DSDS condition afterwards.

To test these hypotheses, we reared *ci* and Actb-SLα:GFP medaka in various conditions and assessed their preferences. First, we transferred two-month-old males (spawning typically starts at three months after hatching) from the separated condition to the mixed condition and let them mature. This breeding provided males the opportunity to associate with females of the different strain prior to mate-choice trials. However, the males strongly preferred females of the same strain (Fig. 4A), as they did when they had been reared in separated conditions (Fig. 2) (Fukamachi et al., 2009a; Utagawa et al., 2016).

We next transferred two-month-old males from the separated condition to the DSDS condition. This breeding forces males to associate and mate with only females of the different strain prior to mate-choice trials. However, the males preferred females of the same strain, which they had never mated with, and mostly ignored females of the different strain, which they had been mating with daily (Fig. 4B).

Then, we put hatched larvae into the mixed condition and transferred them at about two months old to the separated conditions. Again, the males strongly preferred females of the same strain (Fig. 4C). Taken together, associating or mating experiences during either the larval/juvenile or adolescent/adult stages are insufficient in making males interested in females of the different strain.

**Fig. 5.**
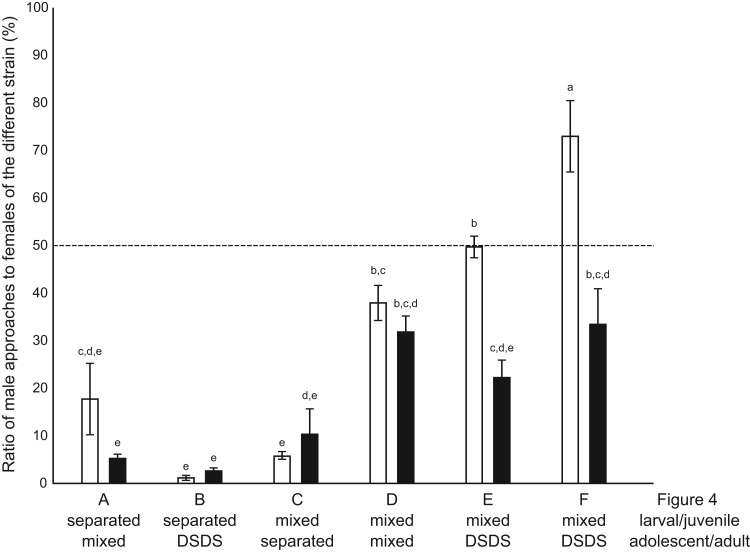
**Summary of data in** **Fig.** **4****.** Ratios of males' approaches to females of the different strains are shown (white, *ci* males; black, Actb-SLα:GFP males). Corresponding figures (Fig. 4A–F) and breeding conditions before/after sexual maturation are indicated below. Letters (a–e) represent significant differences (*P*<0.05) according to an ANOVA and a Tukey's HSD post hoc test. Error bars represent s.e.m.

When males had been reared under the mixed condition during both the larval/juvenile and adolescent/adult stages, however, interest in females of the different strain significantly increased (Fig. 4D; see also Fig. 5), demonstrating that sexual preference could be affected by tank mates. This result supports the third of the above hypotheses. Nevertheless, it simultaneously supports the first and/or second hypotheses, because preferences of the males were still symmetrically biased toward females of the same strain in spite of the identical (i.e. mixed) conditions that the males had experienced.

Consistent results could be obtained when we transferred males from the mixed condition to the DSDS condition; males' interest in females of the different strain was significantly increased (Fig. 4E,F; see also Fig. 5). Interestingly, preference of the *ci* males became not at all biased or even oppositely biased, although such phenomena could not be detected in the Actb-SLα:GFP males. The cause of these exceptionally asymmetrical results between *ci* and Actb-SLα:GFP males is discussed below.

## DISCUSSION

### Genetic factors that influence sexual preference

The *ci* and Actb-SLα:GFP males, which had consistently been bred in mixed conditions, still preferred females of the same strain (Fig. 4D). This result forecasts the existence of an innate factor that makes males prefer females of the same strain, the *SLα* transgene (Fukamachi et al., 2009b). However, the transgene does not always make males prefer Actb-SLα:GFP females; for instance, Actb-SLα:GFP males with the *r* mutation, which reduces xanthophores, preferred *ci* females instead of Actb-SLα:GFP females (Utagawa et al., 2016). Therefore, there seems to be an indirect mechanism in which the *SLα* transgene biases sexual preference; medaka seem to perceive and prefer mates with skin color the same as their own.

In this indirect mechanism, any skin-color gene can potentially influence sexual preference, and the *r* gene has actually been shown to be one of them (Utagawa et al., 2016). However, the *slc45a2* gene that controls melanophores or the *GFP* or *DsRed* transgene that artificially stains the trunk green or red had little effect on sexual preference; i.e. the mutant or transgenic males, which had been bred in separated conditions, did not necessarily prefer females of the same strain (Fukamachi et al., 2009a; Nakao and Kitagawa, 2015; Utagawa et al., 2016). Thus, there seem to be specific colors that medaka perceive as preferable when selecting reproductive partners.

Speciation theories sometimes suppose pleiotropic effects of a gene that controls both sexual trait and sexual preference (Wolf et al., 2010). Although such genes (for instance, a gene that causes exaggerated ornaments to develop and makes neural circuits to prefer the ornaments) sound somewhat unrealistic, our study, probably for the first time by genetic engineering, clearly demonstrated that such a harmoniously pleiotropic gene could exist. The *ci* and Actb-SLα:GFP medaka, which are one mutation (transgene) away from each other and assortatively mate in complete sympatry (Table 1), might represent the initial stage of speciation by mate choice (further discussed below).

### Environmental factors that influence sexual preference

The genetic factors discussed above, however, bias sexual preferences only weakly (see Fig. 4D), as is the case in other animals (Schielzeth et al., 2010). Significant effects apparently come from environmental factors such as surrounding fish (Fig. 5).

Although the sample size in Experiment III is not very large (*n*=8 per breeding condition) and the results may fluctuate, the present study seems to indicate that the sexual preferences of medaka are established gradually during growth and maturation. As shown in Fig. 4D–F, males have increased interest in females of the different strain only when rich opportunities for association/mating with the different strain were provided. Association/mating opportunities during either the larval/juvenile or adolescent/adult stages did not increase interest in the different strain (Fig. 4A–C). These results are similar to those demonstrated over 50 years ago using doves, whereby “not only the early experience during an optimum period but also continued experience throughout the bird's life has an effect on adult behavior” (Klinghammer and Hess, 1964).

Our study provided an additional insight into the adult behavior. Once established, sexual preferences can hardly be altered by sexual experiences (Fig. 2). This finding in a model fish is implicative. If the strongly biased preferences of *ci* and Actb-SLα:GFP medaka can secondarily be relieved or reversed somehow, the methods might be applicable to sexual or reproductive problems in other vertebrates. Our result already indicates that forced association or mating has little effect in this regard (Fig. 2). Further studies on the brain or sensory organs of *ci* and Actb-SLα:GFP medaka would reveal how this continual maintenance could be achieved, for instance, by gene expression, neural circuits, or neural activities.

The asymmetrical preferences between *ci* and Actb-SLα:GFP medaka (Fig. 4E,F) need further investigation. The increased interest by *ci* males may indicate learned preferences by repeated mating with Actb-SLα:GFP females (i.e. a rewarding system) (Tenk et al., 2009); however, such effects were not observed in the Actb-SLα:GFP males. Among many considerable reasons, it is most likely that phenotypes of the presented females might be different in addition to the skin-color difference. That is, the *ci* and Actb-SLα:GFP males, which had established weak skin-color preferences because of the rich opportunities to associate/mate with the different strain (Fig. 4D), might choose females based on other criteria such as body size or susceptibility to spawning.

Indeed, a similar result could be obtained using males that had been bred in a consistently mixed condition. Both the *ci* and Actb-SLα:GFP males, which had not experienced the DSDS condition, significantly preferred larger Actb-SLα:GFP females (body length, 29.3±0.4 mm; body height, 4.4±0.2 mm) to smaller *ci* females (body length, 26.3±0.4 mm; body height, 3.5±0.1 mm) (data not shown). Thus, mating experiences seemed to have only negligible effects on, not only maintenance (Fig. 2), but also the establishment of sexual preferences.

### Sympatric speciation by mate choice alone?

The strongly or completely suppressed gene flow between *ci* and Actb-SLα:GFP medaka in perfect sympatry (Table 1) is intriguing; if this suppression continues for generations, the strains could speciate. As we have shown, their symmetrical, strong, and long-lasting preferences can be established even when they have temporal opportunities for association/mating with the different strain during growth and maturation (Fig. 4A–C). That is, a shoaling preference (Engeszer et al., 2007) or background preference (Bault et al., 2015) may be sufficient for establishing a reproductive barrier. However, such conditions are not sympatric specifically, even when the medaka strains are living in close physical contact. Thus, as some theoretical studies have suggested (Arnegard and Kondrashov, 2004; Kirkpatrick and Nuismer, 2004; van Doorn et al., 2004), our results may provide an empirical example supporting the theory that, in perfect sympatry (Fig. 4D), speciation by mate choice alone is not very likely.

## MATERIALS AND METHODS

### Fish

All experiments were conducted using two medaka strains, *ci* (Fukamachi et al., 2004) and Actb-SLα:GFP (Fukamachi et al., 2009b). All fish hatched, grew, and matured in our laboratory, where light was provided by ordinary fluorescent lamps for 14 h per day (08:30 h∼22:30 h) and breeding water (27°C) was regularly circulated and filtrated. We incubated embryos in Petri dishes (different strains in different dishes) and fed hatched larvae with fine-ground TetraMin (Tetra) and live *Paramecium*. Juveniles and adults were fed with live *Artemia*. All the following experiments have been approved by the Animal Experiment Committees of Japan Women's University.

### Experiment I: the stability test

We prepared mature fish of the *ci* and Actb-SLα:GFP strains, which had been reared separately since hatching. These fish were then kept in either of the following conditions for 12 weeks: (1) the mixed condition where one *ci* male, one Actb-SLα:GFP male, one *ci* female, and one Actb-SLα:GFP female were kept in the same tank, or (2) the different-sex-different-strain (DSDS) condition where two *ci* males and two Actb-SLα:GFP females (or two Actb-SLα:GFP males and two *ci* females) were kept in the same tank. The tank size was 20×13 cm^2^ with a water level of ∼5 cm. During breeding, we tested sexual preferences of the males by mate-choice trials once a week as follows (see also Fig. 1).

A day before the trials, we prepared four tanks, in each of which one *ci* male, one *ci* female, one Actb-SLα:GFP male, and one Actb-SLα:GFP female were placed. The males and females were kept in different compartments using a translucent separator with slits in order to prevent spawning until the next morning.

The following morning, the four tanks were set under a commercial video camera. One of the two males was taken out of each tank, the separators were removed, and the mating behaviors were recorded for 30 min. Then, we replaced the males and continued the recording for another 30 min. Males removed in the first trials were changed weekly. After these eight mate-choice trials (four tanks×two sets), we put all the males back into the tanks and continued mixed or DSDS breeding.

Females often spawned eggs during the first trials, but we left the eggs attached to the cloaca as in previous studies (Fukamachi et al., 2009a; Utagawa et al., 2016). This is because males of *ci* and Actb-SLα:GFP seemed to prefer females of the same strain irrespective of spawning states, for example, they approached spawned females of the same strain much more frequently than yet-to-spawn females of the different strain. This characteristic of males is not always fruitless because females sometimes spawn multiple times a day.

The recorded behaviors were manually analyzed by counting the number of approaches (jerking motions) of a male toward each female (see Fukamachi et al., 2009a for rationales) and the ratio was regarded as male sexual preference in the trial. We ignored all trials in which fewer than 10 approaches could be counted because the ratios could be biased or unbiased by accident. These ratios were used to calculate the mean and the standard error of the mean (s.e.m.) for each strain, condition, and week. We regarded preferences as biased if the s.e.m. (which were nearly equal to 95% confidence intervals; data not shown) did not include 50:50.

### Experiment II: the strength tests

The protocol for Experiment IIa (two-male-one-female trials) is almost identical to that of the one-male-two-female trials described above. The only differences were that we took one female (instead of male) out of each tank for the first and second trials and repeated the trials on four consecutive days by rotationally changing female individuals [i.e. a total of eight females (two females per day) were presented to each competing pair of males]. Video-recorded behaviors were manually analyzed by counting the number of approaches from each male in a trial, which were used to calculate a ratio between the male pair. The mean and s.e.m. of the ratios were also calculated for each male pair and presented strain of female.

Experiment IIb (paternity identification after mass breeding) proceeded as follows. We prepared mature males of *ci* and Actb-SLα:GFP and verified by mate-choice trials (see Experiment III) that each male preferred females of the same strain. Then, six of the males (either six Actb-SLα:GFP or three *ci* plus three Actb-SLα:GFP) were put into a tank (150×100 cm^2^ with a water level of 8 cm) with six *ci* females and were left to mate freely for several days. Spawned eggs were collected every morning and incubated in Petri dishes for embryonic development.

The transgene in Actb-SLα:GFP weakly expresses the reporter GFP (Fukamachi et al., 2009b). Therefore, the embryos become GFP-positive or GFP-negative, when a *ci* female is mated with an Actb-SLα:GFP or *ci* male, respectively. Thus, by observing the presence or absence of GFP fluorescence in each embryo under a SZX16 fluorescence stereomicroscope (Olympus), we identified their paternity.

For statistics, we performed a chi-square test using a null hypothesis that the *ci* females randomly mated with the *ci* and Actb-SLα:GFP males reproducing equal numbers of GFP-positive and GFP-negative embryos.

### Experiment III: the establishment test

Before the sex of the medaka became apparent through secondary sexual characteristics in the dorsal and anal fins (called the larval/juvenile stages in this manuscript), we tested two conditions of breeding. The separated condition, in which different strains were kept in different tanks in a similar density, and the mixed condition, in which different strains were kept in the same tank in similar numbers.

After the sex became apparent at approximately two months after hatching (called the adolescent/adult stages in this manuscript), we kept them in one of the following three conditions: the separated condition, the mixed condition, or the DSDS condition (see Experiment I for the latter two conditions). All the four or more fish in each tank freely associated and mated with tank mates until being used in mate-choice trials.

We conducted mate-choice trials using these fish as described in Experiment I, except that we repeated the trials on four consecutive days by rotationally changing combinations of males and females (Fig. 1). Preference of a male was calculated as an average ratio of valid trials (i.e. with 10 or more approaches). These average preferences were then used to calculate a mean and s.e.m. for each strain and breeding condition. Statistical differences among the conditions were analyzed using an ANOVA and a Tukey's HSD post hoc test.
